# Hypoimmunogenic Human Pluripotent Stem Cells as a Powerful Tool for Liver Regenerative Medicine

**DOI:** 10.3390/ijms241411810

**Published:** 2023-07-22

**Authors:** Piera Trionfini, Elena Romano, Marco Varinelli, Lorena Longaretti, Paola Rizzo, Roberta Giampietro, Annalina Caroli, Sistiana Aiello, Marta Todeschini, Federica Casiraghi, Giuseppe Remuzzi, Ariela Benigni, Susanna Tomasoni

**Affiliations:** Istituto di Ricerche Farmacologiche Mario Negri IRCCS, 24126 Bergamo, Italy; elena.romano@marionegri.it (E.R.); marco.varinelli@marionegri.it (M.V.); lorena.longaretti@marionegri.it (L.L.); paola.rizzo@marionegri.it (P.R.); annalina.caroli@marionegri.it (A.C.); sistiana.aiello@marionegri.it (S.A.); marta.todeschini@marionegri.it (M.T.); federica.casiraghi@marionegri.it (F.C.); giuseppe.remuzzi@marionegri.it (G.R.); ariela.benigni@marionegri.it (A.B.); susanna.tomasoni@marionegri.it (S.T.)

**Keywords:** hypoimmunogenic iPSC, cell therapy, regenerative medicine, endothelial cells, hepatocytes, hepatic stellate cells

## Abstract

Induced pluripotent stem cells (iPSC) have huge potential as cell therapy for various diseases, given their potential for unlimited self-renewal and capability to differentiate into a wide range of cell types. Although autologous iPSCs represents the ideal source for patient-tailored regenerative medicine, the high costs of the extensive and time-consuming production process and the impracticability for treating acute conditions hinder their use for broad applications. An allogeneic iPSC-based strategy may overcome these issues, but it carries the risk of triggering an immune response. So far, several approaches based on genome-editing techniques to silence human leukocyte antigen class I (HLA-I) or II (HLA-II) expression have been explored to overcome the immune rejection of allogeneic iPSCs. In this study, we employed the CRISPR/Cas9 (clustered regularly interspaced short palindromic repeats/CRISPR associated protein 9) system to delete the β2-Microglobulin (*B2M*) and the Class II Major Histocompatibility Complex Transactivator (*CIITA*) genes, essential for the correct surface expression of HLA-I and HLA-II proteins. The resulting hypoimmunogenic iPSC line has a normal karyotype, expresses the pluripotency stem cell markers, and is capable of differentiating into the three embryonic germ layers. Furthermore, we showed that it specifically retains the ability to differentiate towards different liver cells, such as endothelial-like cells, hepatocyte-like cells, and hepatic stellate-like cells. Our results indicate that hypoimmunogenic iPSCs could give a new cost-effective and off-the-shelf opportunity for cell therapy in liver diseases.

## 1. Introduction

Induced pluripotent stem cells (iPSC) represent a great source for different medical research areas, from disease modeling and drug discovery to regenerative medicine.Their self-renewal capacity and their ability to differentiate into any cell type of the human body prompted researchers to generate patient-specific pluripotent stem cells for research and clinical purpose.

From iPSC discovery by Takahashi and Yamanaka in 2006 [1], various strategies have been developed and new ones continue to emerge aiming at optimizing the technology and improving safety and clinical use. Clinical trials using iPSCs are rapidly increasing and they are mainly focused on ocular diseases, malignancies, neural degenerative disorders and cardiovascular diseases [2]. However, the cost of autologous iPSC therapy, due to reprogramming, differentiation, and quality control of patient-specific iPSCs, is not sustainable and forced the research towards the use of allogeneic cells with several efforts trying to solve the immune rejection issue caused by HLA mismatch.

In recent years, researchers have attempted to reduce immunogenicity by genetically modifying pluripotent stem cells using the CRISPR/Cas9 genome editing technology to ablate HLA molecules or related genes. The HLA loci are encoded in a 3600 kb segment of the short arm of the human chromosome 6 (6p21). This is the highest polymorphic region in the human genome, determining the individual genetic variability and the potential to respond to a wide range of infectious pathogens. The HLA system consists of three classes: the class I molecules (HLA-A, HLA-B and HLA-C) are expressed on all nucleated cells, the class II molecules (HLA-DP, HLA-DQ, and HLA-DR) are expressed mostly on antigen-presenting cells (APC), and the class III molecules are components of the complement system, 21-hydroxylase, heat shock protein, and tumor necrosis factors [3].

So far, different approaches have been employed for generating iPSCs with a low immunogenic profile by deleting HLA-I genes directly or targeting some of the genes that are essential for their expression, together with the depletion of HLA-II-related ones. Moreover, different strategies have been explored to overcome the susceptibility of HLA-I-deficient cells to natural killer (NK) cell-mediated lysis [4,5] such as the overexpression of the immunomodulatory factors Programmed Death Ligand-1 (PD-L1), HLA-G, and integrin-associated protein (CD47) [6,7],the HLA-E overexpression concomitant to the PVR cell adhesion molecule (CD155) ablation [8] and the deletion of immunogenic *HLA-A/HLA-B* genes and the retention of the *HLA-C* gene, which has the ability to suppress NK cells [9].

Here, we employed the CRISPR/Cas9 system to generate hypoimmunogenic iPSCs by targeting the *B2M* gene which encodes for the β2M protein required for the folding and the stable surface expression of HLA-I molecules, and the *CIITA* gene, the master regulator of *HLA-II* gene expression. Additionally, we isolated a HLA-I/HLA-II-deficient clone from a commercially engineered CIITA/B2M knockout cell pool (Synthego). We evaluated the self-renewal capacity and the ability to differentiate in three germ-layers of both double knockout (dKO) clones. We found that the hypoimmunogenic iPSC lines can be efficiently differentiated into endothelial cells, hepatocytes, and hepatic stellate cells. Such hypoimmunogenic iPSC-derived populations have normal morphology and functionality in vitro and they could be used as in vitro models to mimic the complex structure and functionality of the liver.

## 2. Results

### 2.1. Generation of CIITA and B2M dKO iPSC Clone

Here, we generated a CIITA/B2M knockout iPSC line by means of two sequential CRISPR/Cas9 editing events in control iPSC (IRFMNi001-B), that have been derived from Normal Human Dermal Fibroblasts Neonatal (NHDF-Neo) using Sendai virus technology. First, we knocked out the *CIITA* gene using a plasmid-based strategy, as recently published in Romano et al., 2021 [10]. The line thus generated (IRFMNi001-B-1) is characterized by a 1 bp insertion on both alleles (c.278_279insA) leading to a truncated nonfunctional CIITA protein (p.A94Gfs*33).

**Figure 1:**
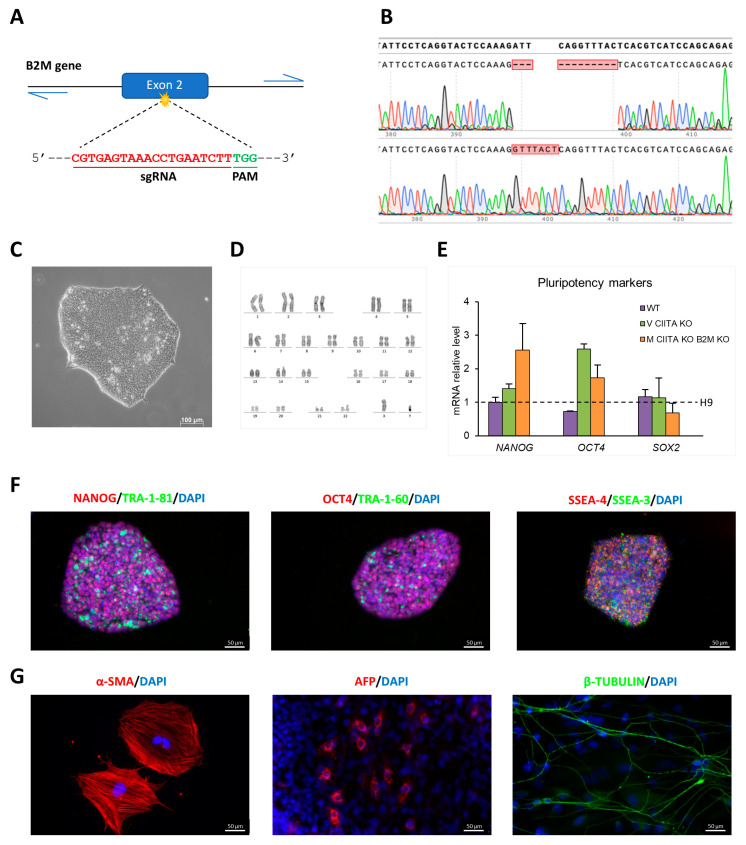
Characterization of M HYPO iPSC line: (**A**) Schematic representation of CRISPR/Cas9 strategy to target the *B2M* gene. (**B**) Chromatogram showing the Sanger sequencing of *B2M* single alleles isolated by TOPO TA cloning and the alignment to the WT sequence using the SnapGene software, confirming the presence of compound heterozygous mutations in the targeted region of *B2M*. (**C**) Bright-field image showing M HYPO cell morphology. Scale bar: 100 μm. (**D**) Karyotype analysis of M HYPO iPSC clone. (**E**) Analysis of transcript levels of the pluripotency marker genes *OCT4*, *NANOG,* and *SOX2* using qRT-PCR. Data were normalized using *GAPDH* and expressed relatively to the H9 cell line. Data are expressed as the mean ± standard deviation (SD) of three independent biological experiments. (**F**) Immunostaining analysis for the pluripotency markers OCT4, NANOG, SSEA-3, SSEA-4, TRA-1-60, and TRA-1-81. Scale bars: 50 μm. (**G**) Immunofluorescence for the endodermal marker α-fetoprotein (AFP), the ectodermal marker βIII-tubulin (β-TUBULIN), and the mesodermal marker α-smooth muscle actin (α-SMA). Scale bars: 50 μm.

To edit the *B2M* gene (NCBI Reference Sequence: NM_004048.3) we took advantage of a CRISPR/Cas9 ribonucleoprotein specific to its second exon (5′-CGTGAGTAAACCTGAATCTT-3′) (Figure 1A). After nucleofection, GFP^+^ iPSCs were sorted using fluorescence-activated cell sorting (FACS) and they were seeded on a mouse embryonic fibroblast (MEF)-feeder layer. Single cells were clonally expanded, analyzed using Sanger sequencing, and the hypothesized deletions were disentangled and verified using the Tracking of Indels by DEcomposition (TIDE) online software tool. Among the colonies, we identified a compound heterozygous *B2M* knockout clone (M HYPO, IRFMNi001-B-2) that was further confirmed through TOPO TA cloning (Figure 1B and Figure S1A). The clone is characterized by a substitution of 2 bp with 6 bp on one allele that causes a reading frame shift leading to a premature stop codon (c.79_80delinsGTTTAC, p.I27Vfs*31). Instead, the other allele shows a 12 bp in-frame deletion (c.79_90delATTCAGGTTTAC) resulting in the deletion of three amino acid residues at the N-terminus (p.dell27-Y30) (Figure S1A). Molecular modelling, obtained using SWISS-MODEL, a fully automated protein structure homology-modelling server [11], shows that the p.delI27-Y30 deletion likely results in the destabilization of the N-terminal domain of β2M (Figure S1B) and it is predicted to be disease-causing by the Mutation Taster Analysis software [12]. 

The presence of mutations in the top six predicted potential off-target sites of Cas9 identified using the online free software Crispor was excluded via Sanger sequencing [13]. The M HYPO clone maintains a stem-cell-like morphology, it has a normal karyotype, and it is able to differentiate spontaneously in vitro into derivatives of the three germ layers (Figure 1C,D,G). Moreover, it expresses the pluripotency markers at mRNA (Figure 1E) and protein level (Figure 1F). The edited clone was authenticated using STR (Short Tandem Repeat) analysis, revealing an identical profile to the parental NHDF-Neo from which the iPSCs were derived.

### 2.2. Differentiation towards Endothelial-like Cells (EC)

**Figure 2:**
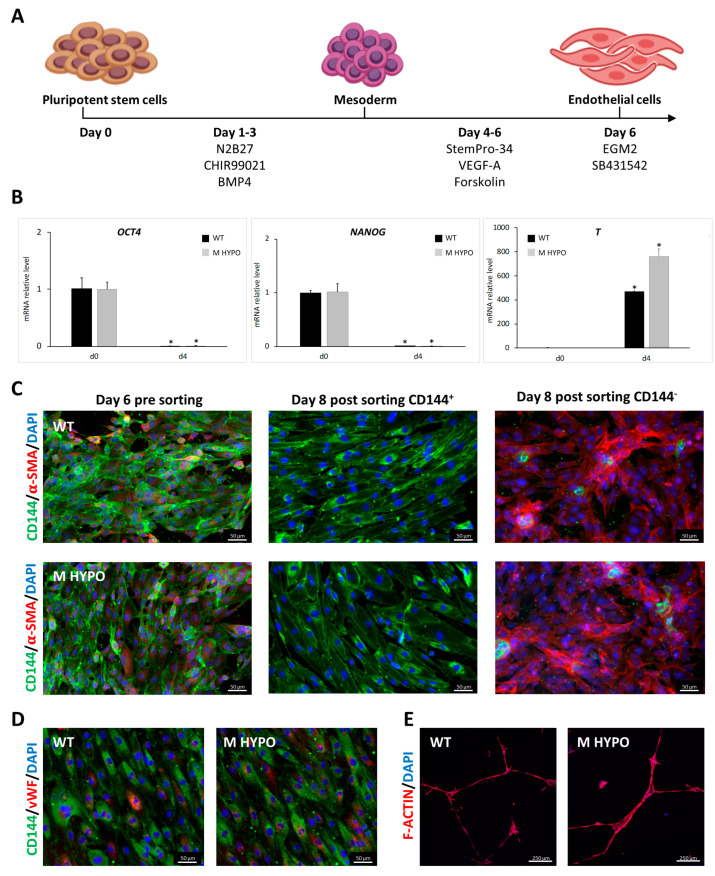
Differentiation of iPSCs towards endothelial-like cells: (**A**) Schematic diagram of the experimental protocol. (**B**) qRT-PCR analysis for pluripotent markers (*OCT4*, *NANOG*) and mesoderm marker (*T*) during EC differentiation. Results were normalized by *HPRT* and expressed relatively to the undifferentiated control (*n*  =  3). Data are means ± SD; * *p* < 0.05 vs. corresponding day 0. (**C**) Representative immunofluorescence images for the expression of CD144 (vascular endothelial cadherin) and α-SMA on EC-WT (upper panels) and EC-M HYPO (lower panels) pre and post sorting. Scale bars: 50 μm. (**D**) Expression of CD144 and vWF (von Willebrand factor) by immunofluorescence of EC-WT (left) and EC-M HYPO (right). Scale bars: 50 μm. (**E**) In vitro tube formation assay of EC-WT (left) and EC-M HYPO (right). Scale bars: 250 μm.

To derive ECs from M HYPO clone (EC-M HYPO) and its isogenic control (EC-WT) we used our optimized two-step differentiation protocol previously described [14] (Figure 2A). The mesodermal specification was achieved using BMP4 (Bone Morphogenetic Protein 4) and CHIR99021, that is a glycogen synthase kinase 3β (GSK-3β) inhibitor and a canonical Wnt pathway agonist. Subsequently, cells were exposed to VEGF-A (Vascular Endothelial Growth Factor A) and Forskolin, a potent activator of adenylyl cyclase, to promote endothelial differentiation. To assess an efficient mesoderm induction in both M HYPO clone and its isogenic control, we confirmed the downregulation of pluripotent stem cell markers, such as OCT4 and NANOG, by day 4 of differentiation and the induction of primitive streak/mesoderm lineage markers, such as T-Brachyury (T) (Figure 2B). 

At the end of the differentiation protocol, we purified iPSC-ECs expressing CD144 (vascular endothelial cadherin), a mature endothelial surface marker, using magnetic-activated cell sorting (MACS). Immunofluorescence analysis confirmed effective isolation of CD144^+^ cells from perivascular α-SMA positive ones (Figure 2C). After expansion, EC-M HYPO and EC-WT cells exhibited a cobblestone-like typical morphology and expressed the endothelial markers CD144 and von Willebrand factor (vWF) as revealed by immunostaining (Figure 2D). Endothelial cells derived from both M HYPO and WT iPSCs are able to form capillary-like structures as assessed by Matrigel tube formation assay indicating that they are functional (Figure 2E). 

### 2.3. Differentiation towards Hepatocyte-like Cells (HLC)

**Figure 3:**
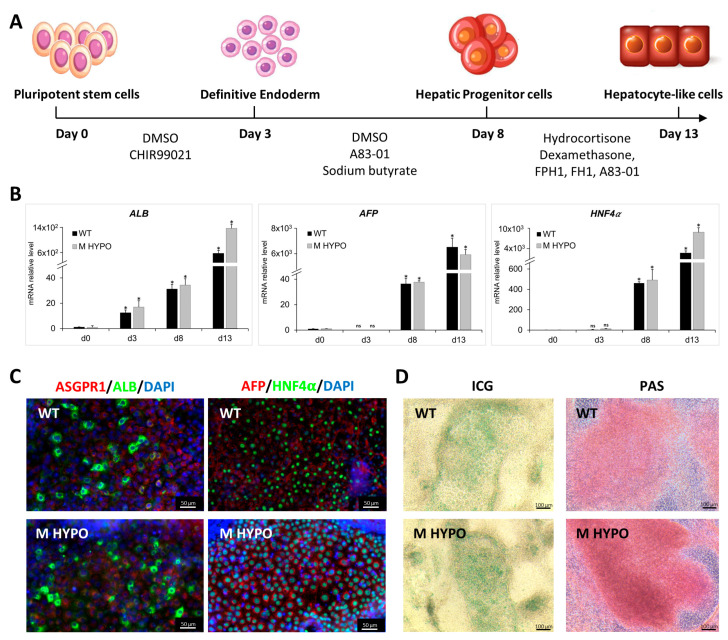
Differentiation of iPSCs towards hepatocyte-like cells: (**A**) Schematic diagram of the differentiation protocol. (**B**) qRT-PCR analysis of hepatic markers *ALB* (Albumin), *AFP,* and *HNF4α* (Hepatocyte Nuclear Factor 4 alpha) on iPSC-HLCs at various differentiation time points. Results were normalized using *HPRT* and expressed as fold versus day 0 (*n* = 3). Data are means ± SD; * *p* < 0.05 vs. corresponding day 0, day 3, and day 8; ns: not significative. (**C**) Immunofluorescence for ASGPR1 (Asialoglycoprotein 1), ALB, HNF4α, and AFP of differentiated cells, HLC-WT (upper panel) and HLC-M HYPO (lower panel). Scale bars: 50 μm. (**D**) Analysis of ICG uptake (left) and PAS staining (right). Scale bars: 100 μm.

To test whether M HYPO cells could give rise to HLCs, they were differentiated using a small-molecules-based differentiation protocol [15], with few modifications. The differentiation protocol recapitulates the developmental stages that occur during embryogenesis in vivo, from the definitive endoderm (DE) stage to hepatic progenitor cells, and finally into hepatocyte-like cells (Figure 3A).

Starting from day 8 of differentiation, we found a significant and progressive upregulation in mRNA expression of genes that encode for both early and late hepatic proteins, such as Hepatocyte Nuclear Factor 4 alpha (HNF4α), α-Fetoprotein (AFP), and Albumin (ALB) (Figure 3B) in HLC-M HYPO and HLC-WT. In both cell lines we confirmed the expression of ALB, Asialoglycoprotein 1 (ASGPR1), AFP, and HNF4α at protein level by immunostaining at day 13 of differentiation (Figure 3C). Additionally, we performed experiments to further test liver-cell-specific functions of the differentiated cells. We used indocyanine green (ICG) as a noninvasive marker of drug uptake and excretion in the liver and we assessed glycogen storage using Periodic Acid-Schiff (PAS) staining in the hepatic cells. At day 13, HLC-M HYPO cells were strongly PAS positive and were able to uptake ICG after only 30 min of incubation, similarly to the isogenic control (Figure 3D). 

### 2.4. Differentiation towards Hepatic Stellate-like Cells (HSC)

**Figure 4:**
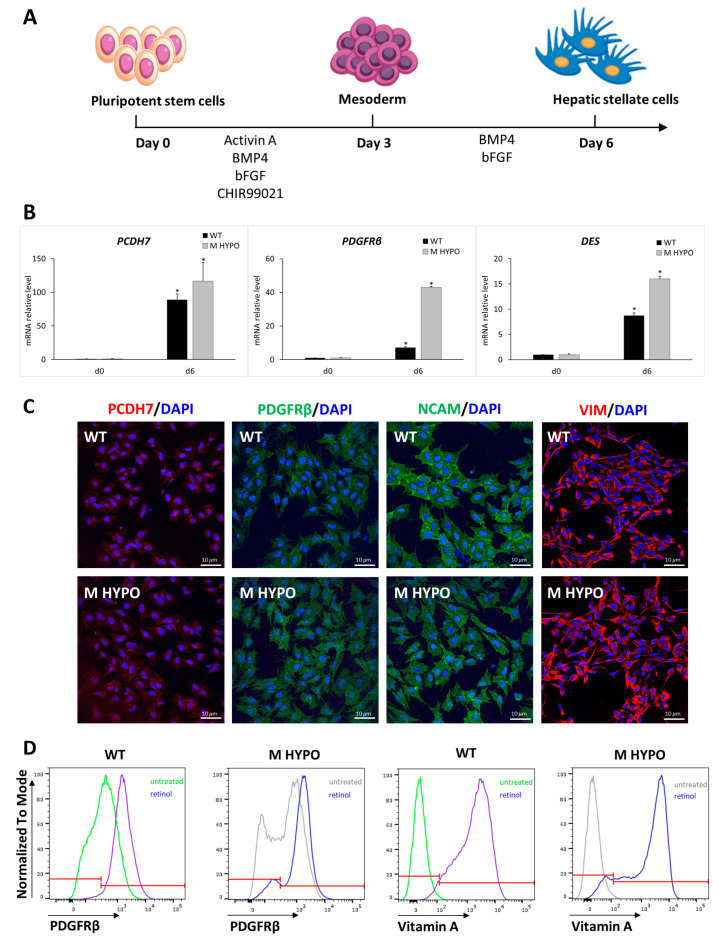
Differentiation of iPSCs towards hepatic stellate-like cells: (**A**) Schematic representation of the differentiation protocol. (**B**) qRT-PCR analysis of HSC markers (*PCDH7*, *PDGFRβ,* and *DES*). Results were normalized to *HPRT* mRNA levels and expressed as fold versus day 0. Data are means ± SD of three independent experiments; * *p* < 0.05 vs. corresponding day 0. (**C**) Immunofluorescence analysis for PCDH7, PDGFRβ, NCAM, and VIM in iPSC-HSCs from WT (upper panel) and M HYPO (lower panel) at the end of the differentiation protocol. Scale bars: 10 μm. (**D**) Representative overlay histogram plots of flow cytometry analysis of iPSC-HSCs showing PDGFRβ-positive cells and vitamin-A-positive cells at the end of a 4-day retinol treatment. The red line divides negative (on the left) from positive (on the right) marker expression.

HSCs are specialized liver pericytes located between hepatocytes and endothelial cells in the space of Disse. They accumulate vitamin A in cytoplasmic lipid droplets and maintain the extracellular matrix homeostasis. We differentiated iPSCs towards an HSC phenotype according to the protocol developed by the group of Miyoshi M. (Figure 4A) [16]. 

For mesodermal induction both M HYPO and WT iPSCs were incubated with BMP4, activin A, bFGF (basic fibroblast growth factor), and CHIR99021 for 3 days. Subsequently, cells were treated with bFGF and BMP4 for another 3 days. At the end of the differentiation protocol (day 6), we assessed the expression of HSC markers. In particular, in both HSC-M HYPO and HSC-WT cells we observed the induction of transcripts specific for HSCs, such as platelet-derived growth factor receptor beta (*PDGFRβ*), protocadherin 7 (*PCDH7*), and desmin (*DES*) (Figure 4B). We also confirmed the expression of the HSCs markers PDGFRβ, PCDH7, vimentin (VIM), and neural cell adhesion molecule (NCAM) using immunostaining (Figure 4C). 

To functionally characterize iPSC-HSCs, we evaluated their ability to store vitamin A by measuring the autofluorescence after UV light excitation. Day-6 differentiated cells were treated for 4 days with retinol and palmitate in order to stimulate vitamin A accumulation in iPSC-HSC lipid droplets. As shown in Figure 4D, after stimulation the cells maintained the expression of PDGFRβ and, as expected, the percentage of vitamin-A-positive cells increased, reaching a mean of ~90%, both in control and double-knockout cells, indicating that *B2M* and *CIITA* gene editing does not affect the ability of HSC differentiated cells to store vitamin A in the cytoplasm.

### 2.5. Assessment of Hypoimmunogenicity of M HYPO EC Cells In Vitro

**Figure 5:**
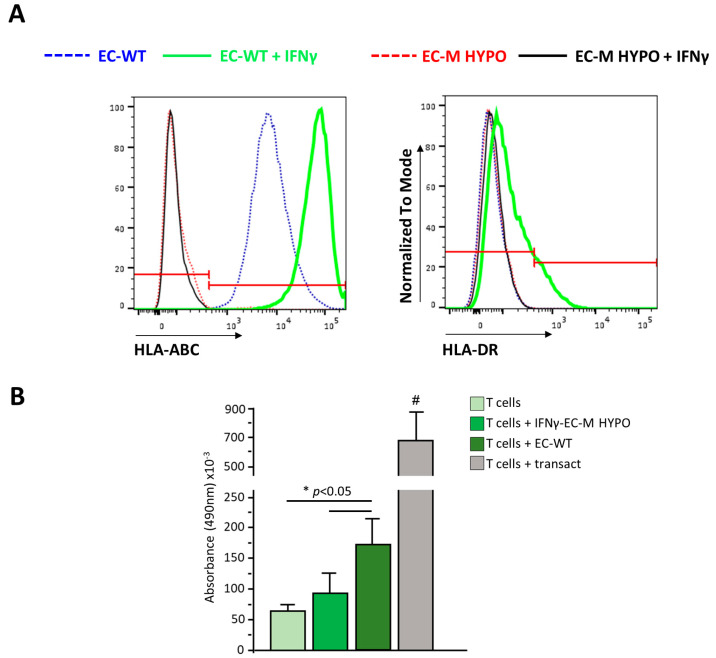
(**A**) Representative overlay histogram plots of flow cytometric analysis of HLA-ABC and HLA-DR expression in untreated and IFNγ-treated endothelial cells derived from WT or M HYPO cells. The red line divides negative (on the left) from positive (on the right) marker expression. (**B**) Proliferation of T cells in resting condition, after coculture with endothelial cells derived from WT or M HYPO iPSCs or after stimulation with Transact, as positive control. # *p* < 0.05 vs. all. Data are means ± SD of three independent experiments.

To confirm that the ablation of both *B2M* and *CIITA* results in the lack of surface expression of HLA-I and HLA-II, respectively, we differentiated both WT and dKO clones into endothelial cells, the most immunogenic cell type among differentiated HLCs, HSCs, and ECs. Moreover, iPSC-ECs were treated with IFNγ (Interferon-gamma) to boost HLA expression. Flow cytometry analysis revealed that untreated EC-WT cells were positive for HLA-I expression and did not express HLA-II. IFNγ treatment induced a strong upregulation of HLA-I and the expression of HLA-II in EC-WT cells. By contrast, HLA-I and HLA-II expression was undetectable both in untreated and in IFNγ-treated EC-M HYPO cells (Figure 5A). These data indicate that the absence of *B2M* and *CIITA* completely translates into the ablation of HLA-I and HLA-II surface expression. 

We performed a T cell proliferation assay to evaluate whether EC-M HYPOs stimulate allogeneic T cells; IFNγ-treated EC cells derived from WT or M HYPO iPSCs were cocultured with allogeneic T cells for 72 h and the proliferation of T cells was assessed using MTS assay. As shown in Figure 5B, at variance with T cells cocultured with IFNγ-stimulated ECs derived from WT iPSCs, the proliferation of T cells induced by EC-M HYPO cells was similar to that of T cells cultured in the resting condition, indicating that they were not able to immune-stimulate T cells.

### 2.6. Analysis of CD47 Expression in Undifferentiated and Differentiated M HYPO Cells

**Figure 6:**
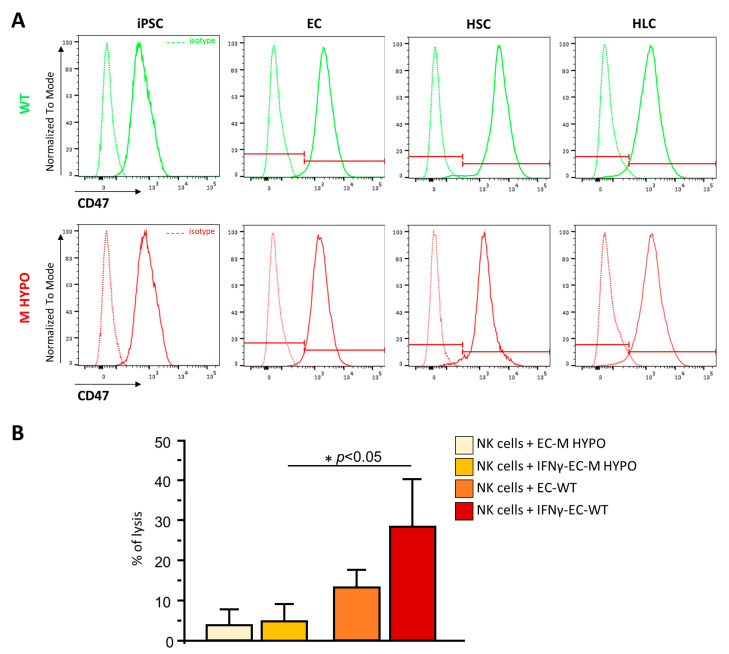
(**A**) Representative overlay histogram plots of flow cytometric analysis of CD47 expression in undifferentiated and differentiated WT (green) and M HYPO (red) iPSCs, and the respective isotype controls (dashed line). The red line divides negative (on the left) from positive (on the right) marker expression. (**B**) LDH release assay after allogeneic natural killer coculture with EC-M HYPO and EC-WT cells treated or not with IFNγ. Data are means ± SD of three independent experiments. * *p* < 0.05 vs. corresponding WT.

In order to avoid the NK-mediated clearance of cells that do not express HLA-I, several strategies have been exploited, including the overexpression of CD47, a ubiquitous membrane protein with a key role in preventing NK cytotoxic response and phagocytosis by macrophages and dendritic cells. We analyzed the surface expression of CD47 in our cells differentiated from WT and M HYPO iPSCs using FACS analysis. As shown in Figure 6A, CD47 expression is already high in both undifferentiated WT and M HYPO iPSCs and its expression did not change once differentiated towards endothelial, hepatic stellate, and hepatocyte-like cells. In light of these findings, we reasoned that we could avoid overexpressing CD47, a common strategy used by many researchers to compensate for the loss of HLA-I [6,7]. To confirm that basal CD47 expression is sufficient to prevent NK cell killing, we evaluated the human allogeneic NK cell cytotoxicity toward differentiated iPSC-ECs using LDH (Lactate dehydrogenase) release assay during 24 h of coculture. As shown in Figure 6B, hypoimmunogenic cells were less sensitive to NK-mediated cytotoxicity, compared to their WT controls, both in unstimulated and IFNγ-stimulated conditions.

### 2.7. Establishment of CIITA and B2M dKO iPSC Clone from a Commercially Gene-Edited Cell Pool

Since the reproducibility of iPSC-based experiments is greatly affected by genetic variability between donor individuals [17], by donor-specific epigenetics retained after reprogramming, and by donor sex and age [18], we generated a second double-knockout clone starting from a different donor. We took advantage of Synthego’s service which performed a two-step knockout strategy using Sp-Cas9 ribonucleoprotein complexes on 802-30F iPSCs (RPChiPS8023G1) sourced from primary human endothelial progenitor cells of a control female donor. The following guides were chosen to knockout, respectively, *CIITA* and *B2M* genes: CAUCGCUGUUAAGAAGCUCC, targeting the second exon of *CIITA*; CAGUAAGUCAACUUCAAUGU, targeting the second exon of *B2M*. We isolated 30 different clones from the double-knockout cell pool using the limiting dilution method. The *B2M* and *CIITA* on-target loci were sequenced for each clone. Among these clones, we selected clone 19L (19L HYPO), that is compound heterozygous for both *B2M* (I allele: c.164_173delTTGAAGTTGA, p.I55Tfs*3; II allele: c.164_169delTTGAAG, p.delE56V57) and *CIITA* (I allele: c.107_108insA, L37Afs*3; II allele: c.108_135delGCTTCTTAACAGCGATGCTGACCCCCTG, E36Dfs*70) (Figure S2A,B). Once we confirmed the normal karyotype of the new hypoimmunogenic clone, we verified its stemness both at the mRNA and protein level, as well as its capacity to differentiate into the three germ layers (Figure S2C–G). Similarly to clone M HYPO, the 19L HYPO clone differentiated successfully towards functional endothelial-like cells upon which we confirmed the absence of HLA-I and HLA-II surface expression and the high basal expression of CD47 as its isogenic control (Figure S3A–C). Moreover, functional hepatocyte-like cells (Figure S3D,E) and hepatic stellate-like cells were obtained from clone 19L HYPO too (Figure S3F,G). 

## 3. Discussion

In this study we have generated hypoimmunogenic iPSCs from two different sources, neonatal fibroblasts and endothelial progenitor cells. We have demonstrated that these cells, besides evading immune rejection, can develop into functional cells of the liver, like hepatocytes and nonparenchymal cells including endothelial and hepatic stellate cells. In particular, hepatocytes were differentiated via endodermal fate from iPSCs while both endothelial and hepatic stellate cells from mesoderm, recapitulating specific in vivo developmental signaling pathways. Double gene editing of *CIITA* and *B2M* and the resulting lack of expression of HLA-I and HLA-II proteins did not affect any of these developmental routes. Furthermore, adult cells derived from HYPO iPSCs exhibited specific functional attributes: EC HYPO cells formed capillary-like structures, HSC HYPO cells accumulated vitamin A in the form of retinyl esters, and HLC HYPO cells expressed albumin, took up ICG, and stored glycogen. These cells could mediate the reconstruction of hypoimmunogenic liver sinusoids in vitro paving the way for regenerative medicine in liver diseases. Chronic liver diseases, characterized by a massive loss of hepatocyte function, and several inborn metabolic diseases require liver transplantation that is limited by the shortage of organs [19,20]. Currently, primary cryopreserved hepatocytes are the gold standard for liver cell therapy [21], although they are characterized by low cell renewal, loss of their phenotypic characteristics and functions once isolated, and poor engraftment in the host liver. In order to overcome these issues, iPSCs are widely explored as an alternative source of human hepatocyte-like cells, thanks to their unlimited proliferation capacity without loss of potency. To date, there are also many attempts of tissue engineering aiming at reconstructing or regenerating liver tissues by combining different liver specific cells derived from iPSCs [22]. Despite being very promising, it is impractical to generate autologous iPSCs and derivatives, because it is time consuming and costly. Instead of generating patient-specific iPSCs, the use of allogeneic cell therapy is preferred, since it enables an industrially scalable generation of high-quality cells at a lower manufacturing cost and it can be available in acute medical situations. This is of utmost importance for inborn liver diseases since allogeneic tissues from healthy nonself-donors are free of disease-causing mutations. The greatest drawback of using allogeneic cells is the immune response they trigger in the recipient, which leads to their rejection. To overcome their immunogenicity, several research efforts are focused on generating an unlimited cell source of hypoimmunogenic iPSCs modulating the expression of proteins involved in immune recognition and in immune suppression using genome engineering approaches [23]. Here, we ablated the human leukocyte antigen class I and class II proteins, using the CRISPR/Cas9 system, to prevent the cytotoxic CD8^+^ T cell and the helper CD4^+^ T cell responses. We confirmed their reduced immunogenicity by demonstrating that they did not elicit allogeneic T cell proliferation in the coculture condition. Moreover, we also showed that our cells were not more sensitive to NK cell killing than WT cells. Previous evidence showed that the ablation of B2M prevents the surface expression of both nonclassical HLA-E, which is required to maintain NK cell tolerance by binding to the inhibitory receptor NKG2A, and of HLA-C molecules, which are ligands for inhibitory killer immunoglobulin-like receptors (KIR) on NK cells [24]. However, despite the absence of HLA-I and the consequent defects in these inhibitory signals, the HYPO cells we generated did not show increased susceptibility to NK cell killing. We found that they displayed a high expression of CD47, a receptor that inhibits NK-mediated phagocytosis by binding the signal regulatory protein α (SIRPα). The high CD47 expression likely counterbalanced the absence of the inhibitory signals, protecting the HLA-I deficient cells via NK-mediated killing. In support of our data, an elegant study by Deuse demonstrated that the CD47-SIRPα axis transmits threshold-dependent inhibitory signals capable of prevailing the stimulatory signals provided by the absence of HLA-I [25]. Moreover, similarly to our findings, Merola found that endothelial cells derived from committed progenitors and knockout for both *B2M* and *CIITA* do not activate or elicit cytotoxic activity from allogeneic NK cells [26]. In the future, we will further confirm the hypoimmunogenicity of our gene-edited clones using immunodeficient mice reconstituted with human T and NK cells whose use is mandatory in preclinical tests [27]. 

In conclusion, we have successfully generated a pool of hypoimmunogenic cells that can be utilized for engineering universal vascularized liver grafts in regenerative medicine. Besides liver diseases, these cells can also be exploited for all those diseases where autologous iPSC-based cell therapy has already been proven to be effective, such as neural diseases, heart diseases, and malignancies, as soon as a reliable differentiation protocol has been developed towards the curative adult cells [28]. 

## 4. Materials and Methods

### 4.1. iPSC Culture

We used iPSCs derived from Normal Human Dermal Fibroblasts Neonatal (NHDF-Neo, Lonza, Milan, Italy) using the Sendai virus technology (https://hpscreg.eu—IRFMNi001-B, released on 17 September 2020) and its subclone knockout for CIITA that we had recently generated using the CRISPR/Cas9 system (https://hpscreg.eu—IRFMNi001-B-1, released on 25 June 2021). The cells were routinely cultured on Matrigel-coated dishes (Matrigel hESC-Qualified Matrix, Corning, Sial S.r.l., Rome, Italy) in mTeSR Plus medium (Stemcell Technologies, Cologne, Germany), detached using Accutase (Gibco, Thermo Fisher Scientific, Waltham, MA, USA) twice a week, and plated at a density of 20,000–60,000 cells/cm^2^. On the day of seeding, 10 µM Rock inhibitor Y-27632 (Sigma–Aldrich, St. Louis, MO, USA) was added to the culture medium. Upon arrival, the CIITA and B2M dKO cell pool purchased from Synthego was cultured in mTeSR Plus. No precoating was required, but iMatrix-511 (Reprocell, Glasgow, Scotland, UK) was added directly to the medium according to the supplier’s recommendations. We isolated single cells via limiting dilution and generated monoclonal populations following the Synthego’s Limiting Dilution and Clonal Expansion protocol. Once the iPSC clones were assessed, they were switched to Matrigel-coated dishes and expanded in mTeSR Plus. All cell lines were routinely tested for mycoplasma contamination, using a N-GARDE Mycoplasma Detection Polymerase Chain Reaction (PCR) Kit (Euroclone, Milan, Italy).

### 4.2. Human iPSC CRISPR/Cas9-Mediated Genome Editing

For B2M knockout, 10^6^ CIITA knockout iPSCs were nucleofected with 10 µg (52 pmol)-Cas9-GFP-protein (Sigma-Aldrich, St. Louis, MO, USA) precomplexed with 390 pmol sgRNA *B2M* custom (Sigma-Aldrich, St. Louis, MO, USA) (RNP:sgRNA = 1:7.5) according to the manufacturer’s instructions. Nucleofection was conducted using the A023 program with the Human Stem Cell Nucleofector Kit 2 and Nucleofector2b Device (Lonza, Milan, Italy). Transfected cells were plated on Matrigel-coated 6-well plates in mTeSR Plus with 10 μM Y-27632. After 24 h, GFP^+^ single cells were isolated through cell sorting (FACSAria IIu; Becton Dickinson Italia S.p.A., Milan, Italy), plated on MEF feeder-coated plates with a density of 500 cells/cm^2^ and then the single-cell-derived colonies were expanded for another 8 days. Thirty emergent clones were picked manually and plated on Matrigel-coated wells in mTeSR Plus medium.

### 4.3. Genotyping and Sanger Sequencing

Genomic DNA was extracted from cells using the DNeasy Blood and Tissue kit (Qiagen, Milan, Italy). The *B2M* and *CIITA* on-target loci were amplified by PCR using TaKaRa LA Taq DNA Polymerase (Takara Bio, Saint-Germain-en-Laye, France) and the primers listed in Table S1. The PCR products were Sanger-sequenced using a BigDye Terminator v3.1 sequencing kit on the 3730 DNA Analyzer (Applied Biosystems, Foster, CA, USA). Clones with hypothesized deletions were disentangled and verified using the Tracking of Indels by Decomposition (TIDE) online software tool (https://tide.nki.nl/, accessed on 1 June 2022). The pathogenicity of the deletions was predicted using Mutation Taster (https://www.mutationtaster.org/, accessed on 1 June 2022). Similarly, mutations in the top six predicted potential off-target sites of Cas9, identified using the online free software Crispor (http://crispor.tefor.net/, accessed on 1 February 2022), including all those that fell within protein coding regions or next to intronic–exonic junctions (<1000 bp), were excluded via Sanger sequencing (Table S1). In both the on- and off-target loci, we looked for the presence of parental clone-specific heterozygous SNPs, which allowed us to be confident to have amplified both alleles ruling out the occurrence of allelic dropout. Molecular modelling was performed with the fully automated protein structure homology-modelling server SWISS-MODEL (https://swissmodel.expasy.org/, accessed on 1 June 2022).

### 4.4. Topo TA Cloning

The *B2M* and *CIITA* on-target loci were PCR amplified from the genomic DNA extracted from the gene-edited cells and the amplicon flanking the CRISPR/Cas9-targeted sites was cloned into the pCR4-TOPO TA cloning vector of the TOPO TA Cloning Kit for Sequencing (K4575J10, Invitrogen, Thermo Fisher Scientific, Waltham, MA, USA). The recombinant vector was transformed into One Shot TOP10 Chemically Competent *E. coli* (Invitrogen, Thermo Fisher Scientific, Waltham, MA, USA) and at least 10 colonies were sequenced using the provided sequencing primers. The sequences were analyzed with the SnapGene software (from Dotmatics; available at snapgene.com).

### 4.5. RNA Extraction and Quantitative Real-Time PCR

Total RNA was isolated using Trizol Reagent (Invitrogen, Thermo Fisher Scientific, Waltham, MA, USA) and treated with DNAse (Promega, Madison, WI, USA), as described by the manufacturer. The RNA (2 μg) was reverse transcripted using the SuperScript VILO cDNA Synthesis kit (Invitrogen, Thermo Fisher Scientific, Waltham, MA, USA), following the manufacturer’s instructions. Quantitative Real-Time PCR assays were performed using SYBR Green PCR Master Mix with primers or Taqman PCR Master Mix with predesigned Taqman probes for the genes of interest according to the supplier’s recommendations (Applied Biosystems, Foster, CA, USA) (Table S2). Gene expression levels were normalized to the housekeeping gene *HPRT*.

### 4.6. Immunofluorescence Staining

Cells were fixed using phosphate-buffered saline (PBS) containing 4% paraformaldehyde (PFA, Thermo Fisher Scientific, Waltham, MA, USA) for 15 min at room temperature (RT). After three washes with PBS, the cells were permeabilized with 0.3% Triton X-100 (Sigma-Aldrich) for 10 min at RT (when necessary) and then incubated with 5% bovine serum albumin (BSA, Sigma-Aldrich, St. Louis, MO, USA) as blocking solution for 1 h. The samples were stained with primary antibodies diluted in 2% BSA solution overnight at 4 °C, followed by the corresponding AlexaFluor-546- or AlexaFluor-488-conjugated secondary antibodies for 1 h at RT. F-actin filaments were stained with TRITC-conjugated phalloidin (Invitrogen, Thermo Fisher Scientific, Waltham, MA, USA). Nuclei were counterstained with 4′,6-diamidino-2-phenylindole (DAPI, Sigma-Aldrich, St. Louis, MO, USA). Images were taken using an inverted confocal laser-scanning microscope (Leica TCS SP8, Leica Microsystems, Milan, Italy) or the Axio Observer Z1 fluorescence microscope (Carl Zeiss, Oberkochen, Germany). The primary antibodies used are listed in Table S3.

### 4.7. Embryoid Body Formation, Karyotype and Cell Line Authentication

Embryoid body formation was performed following the procedure described by Romano E. [10].

Karyotyping was performed in collaboration with the Genetic Medicine Laboratory of the Azienda Socio Sanitaria Territoriale Papa Giovanni XXIII (Bergamo, Italy). Metaphase spreads were prepared after 3 h of treatment with Colcemid (Sigma–Aldrich, St. Louis, MO, USA) and processed for karyotype analysis. A minimum of 20 metaphases were analyzed. Cell-line authentication was performed by ATCC’s Human Short Tandem Repeat (STR) Profiling Cell Authentication Service.

### 4.8. Differentiation of iPSCs into Endothelial-like Cells

To obtain endothelial cells, iPSCs were exposed to a differentiation protocol, as previously described by Ciampi. iPSCs were dissociated using Accutase and plated on growth-factor-reduced Matrigel-coated dishes (Becton Dickinson Italia S.p.A., Milan, Italy) at a density of 30,000–35,000 cells/cm^2^ in mTeSR Plus medium with 10 μM ROCK inhibitor Y-27632. After one day, the medium was replaced with an induction medium consisting of a 1:1 mixture of DMEM/F-12 (Dulbecco’s Modified Eagle Medium F12, Gibco, Thermo Fisher Scientific, Waltham, MA, USA), plus Glutamax (Gibco, Thermo Fisher Scientific, Waltham, MA, USA), and Neurobasal medium with N2 and B27 supplements (Gibco, Thermo Fisher Scientific, Waltham, MA, USA), in the presence of 10 μM CHIR99021 (Miltenyi Biotec S.r.l., Bologna, Italy) and 25 ng/mL BMP4 (Gibco, Thermo Fisher Scientific, Waltham, MA, USA). The induction medium was maintained for 3 days and thereafter replaced by StemPro-34 medium (Gibco, Thermo Fisher Scientific, Waltham, MA, USA) supplemented with 200 ng/mL VEGF-A (Gibco, Thermo Fisher Scientific, Waltham, MA, USA) and 2 μM Forskolin (Sigma–Aldrich St. Louis, MO, USA) for 2 more days in order to induce the endothelial phenotype. At day 6 of differentiation, cells were dissociated using Accutase and sorted using MACS separation to obtain pure endothelial cells using CD144 antibody conjugated with microbeads (Miltenyi Biotech S.r.l., Italy); 20 μL for a total of 10^7^ cells) and LS MACS columns according to the manufacturer’s instructions. The CD144^+^ cells were cultured on fibronectin-coated (2 μg/cm^2^, Becton Dickinson Italia S.p.A., Milan, Italy) dishes in StemPro-34 medium supplemented with 50 ng/mL VEGF-A. For expansion, CD144^+^ cells were grown in EGM-2 medium (Endothelial Cell Growth Medium-2, Lonza, Milan, Italy) without hydrocortisone, supplemented with 20% defined fetal bovine serum (FBS HyClone, Euroclone, Milan, Italy) and 10 μM SB431542 (Sigma–Aldrich St. Louis, MO, USA) (EC expansion medium). All experiments described in the manuscript were performed using iPSC-derived ECs between passages three and five. 

### 4.9. Tube Formation Assay

To measure the capillary-like formation of iPSC-ECs, we performed tube formation experiments. A volume of 120 μL of growth-factor reduced basement membrane Matrix Matrigel (GFR, Corning, Sial S.r.l., Rome, Italy) was added per well of an 8-well cell culture chamber slide (Falcon) on ice and then incubated for 1 h at 37 °C to allow the gel to solidify. A total of 5 × 10^3^ ECs per well were then seeded onto the matrix and cultured in EC expansion medium. Then, 18 h later, the formed tubes were fixed using 4% PFA and stained with Phalloidin-Rhodamin (Thermo Fisher Scientific, Waltham, MA, USA) to visualize F-actin and DAPI. Images were acquired as Z-stacking with an inverted confocal laser-scanning microscope, and visualized at a maximum projection of about 60 optical slices. 

### 4.10. Differentiation of iPSCs into Hepatocyte-like Cells

Hypoimmunogenic iPSCs were exposed to a small-molecules-based differentiation protocol, as described by Du [15], with few modifications. The iPSCs were seeded at a density of 200,000 cells/well in Matrigel-coated 6-well plates in mTeSR Plus medium. For initial definitive endoderm induction, the medium was changed to 0.5% DMSO (Dimethyl sulfoxide) in mTeSR Plus medium and after 24 h switched to RPMI 1640 with B27 Supplement Minus Insulin (Gibco, Thermo Fisher Scientific, Waltham, MA, USA), along with 3 μM CHIR99021. At day 3, CHIR99021 was withdrawn and the cells were treated with RPMI 1640/B27 basal medium alone for another 24 h. To induce hepatic specification, the cells were cultured in Advanced F12 basal medium (95% Advanced DMEM/F-12 medium, 1% B-27 Serum-Free Supplement, 1% KnockOut Serum, 1% Gluta-MAX Supplement, 1% Non-Essential Amino Acids Solution, and 1% penicillin/streptomycin, Gibco, Thermo Fisher Scientific, Waltham, MA, USA) with 0.5 μM A83–01 (Sigma–Aldrich St. Louis, MO, USA), 250 nM sodium butyrate (Sigma–Aldrich St. Louis, MO, USA), and 0.5% DMSO for 5 days. In the last stage, the differentiation medium was switched to Advanced F12 basal medium supplied with commercial small molecules, containing 15 μM FH1 (Sigma–Aldrich St. Louis, MO, USA), 15 μM FPH1 (Sigma–Aldrich St. Louis, MO, USA), 0.5 μM A83-01, 100 nM dexamethasone (Sigma–Aldrich St. Louis, MO, USA), and 10 μM hydrocortisone (Sigma–Aldrich St. Louis, MO, USA) for 5 days.

### 4.11. Differentiation of iPSCs into Hepatic Stellate-like Cells

To obtain HSCs, iPSCs were exposed to a differentiation protocol, as previously described by Miyoshi [16]. iPSCs were seeded at a density of 60,000 cells/cm^2^ in a 12-well plate coated with growth-factor reduced Matrigel and maintained in mTeSR Plus medium. The day after, cells were cultured in differentiation medium (DMEM/F12 medium plus 2% B-27 supplement, 1% N2 supplement) supplemented with 10 μM CHIR99012, 30 ng/mL BMP4, 10 ng/mL activin A (Gibco, Thermo Fisher Scientific, Waltham, MA, USA), and 10 ng/mL bFGF (Tebu Bio, Milan, Italy) for 3 days. From day 4 to 6, cells were treated with 50 ng/mL BMP4 and 100 ng/mL bFGF. For vitamin A detection, at day 6 of the differentiation protocol the iPSC-HSCs were detached using Trypsin (Gibco, Thermo Fisher Scientific, Waltham, MA, USA) and seeded on fibronectin in RPMI medium plus 2% B-27 supplement, 5 μM retinol (Sigma–Aldrich, St. Louis, MO, USA), and 100 μM palmitic acid (Sigma–Aldrich, St. Louis, MO, USA) for 4 days.

### 4.12. Flow Cytometry

Cells were incubated for 30 min at 4 °C with the following antibodies (Becton Dickinson Italia S.p.A., Milan, Italy): PE Mouse IgG1, k Anti-Human HLA-ABC (clone G46-2.6); BV605 Mouse IgG2a, k Anti-Human HLA-DR (clone G46-6); PE mouse IgG1, k anti human CD47; PE mouse IgG1, k isotypecontrol;PE mouse Anti-Human CD140b IgG2a, k; PE mouse IgG2a k isotype control. Vitamin A storage was analyzed measuring the autofluorescence after UV light excitation (retinyl esters autofluorescence at 328 nm). Cell fluorescence was acquired using FACS LSR Fortessa X-20 (Becton Dickinson Italia S.p.A., Milan, Italy) and the flow data obtained were analyzed using FlowJo software (version 10.7.1—BD Biosciences).

### 4.13. T Cell Proliferation Assay 

iPSC-ECs were treated for 48 h with IFNγ. Then, 48 h later, cells were detached and 40,000 cells were seeded in a U bottom 96-well plate together with T cells (100,000) in a 1/2.5 ratio in RPMI supplemented with 10% human AB serum, human IL-2 (10 U/mL, Miltenyi Biotec, S.r.l., Bologna, Italy), and antihuman CD28 (4 μg/mL, clone CD28.2, Invitrogen, Thermo Fisher Scientific, Waltham, MA, USA). In parallel, T cells isolated from PBMC using magnetic beads (untouched panhuman T cell solation kit, Miltenyi Biotec, S.r.l., Bologna, Italy) were either cultured alone or stimulated using the T cell activator TransAct (Miltenyi Biotec, S.r.l., Bologna, Italy). After 72 h of coculture, T cell proliferation was assessed using a 3-(4,5-dimethylthiazol-2-yl)-5-(3-carboxymethoxyphenyl)-2-(4-sulfophenyl)-2H-tetrazolium (MTS) colorimetric assay (CellTiter96 Aqueous One Solution Cell Proliferation, Promega, Madison, WI, USA) according to the manufacturer’s instructions. In particular, MTS tetrazolium compound (final concentration of 0.5 mg/mL) was added for 2 h at 37 °C. The amount of soluble formazan produced by a reduction of MTS by viable cells was determined by measuring the absorbance at 490 nm using a microplate reader (Infinite m200-pro, Tecan Group Ltd., Männedorf, Switzerland). Background 490 nm absorbance given by the medium alone was subtracted from the average absorbance of cultured cells.

### 4.14. NK Cell-Killing Assay

iPSC-ECs were treated or not for 48 h with IFNγ. Then, 48 h later, cells were detached and 40,000 cells were seeded in a U bottom 96-well plate together with NK cells, isolated using the NK isolation kit (Miltenyi Biotec, S.r.l., Bologna, Italy) in a 1/1 ratio in RPMI supplemented with 10% human AB serum. After 24 h of coculture, cell lysis was assessed using the CytoSelect LDH Cytotoxicity Assay kit (Cell Biolabs Inc., San Diego, CA, USA) according to the manufacturer’s instructions. Results were expressed as percentages of specific NK-induced cell lysis, considering the LDH release obtained via cell incubation with Triton X-100 solution as the maximal cell lysis. 

### 4.15. Statistical Analysis 

Data were reported as mean ± SD. The normality of value distribution was assessed using the D’Agostino–Pearson’s test. Comparisons of three or more groups were performed using one-way analysis of variance followed by a post hoc Student–Newman-Keuls test or Kruskal–Wallis test followed by a post hoc Conover test, as appropriate. Statistically significant differences were assumed at a 5% level of probability. All calculations were made using MedCalc 10.0.1 statistical software (MedCalc Software).

## Data Availability

Not applicable.

## Supplementary Materials

The supporting information can be downloaded at https://www.mdpi.com/article/10.3390/ijms241411810/s1.
