# Supplementary material for: Hypoimmunogenic Human Pluripotent Stem Cells as a Powerful Tool for Liver Regenerative Medicine
Source: Int J Mol Sci. 2023 Jul 22;24(14):11810. doi: 10.3390/ijms241411810 (PMC10380710; doi:10.3390/ijms241411810)
Supplement: Supplementary file 1 [file ijms-24-11810-s001.zip › ijms-2466980-supplementary.pdf]

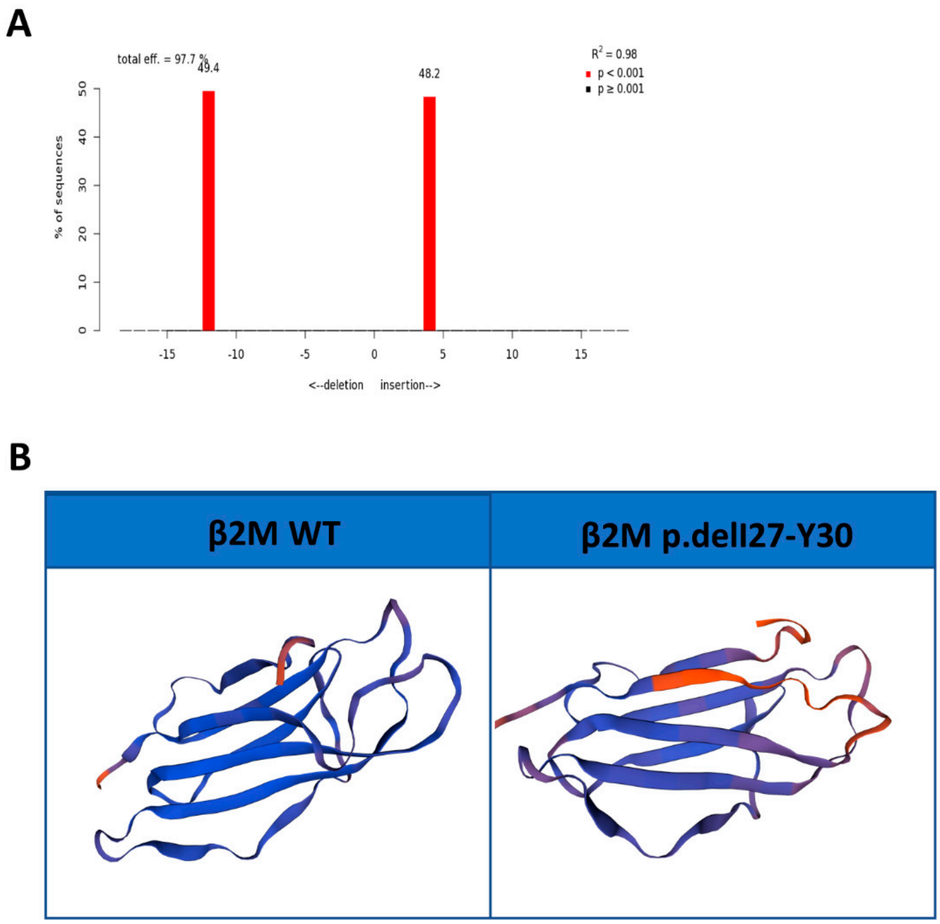

Figure S1. Analysis of *B2M* on-target locus and protein outcome in M HYPO clone: (**A**) Analysis of *B2M* on-target locus using the Tracking of Indels by Decomposition online tool. (**B**) Molecular modelling of β2M protein (P61769) with the p.delI27-Y30 deletion as a consequence of the indel mutation following CRISPR/Cas9 gene editing.

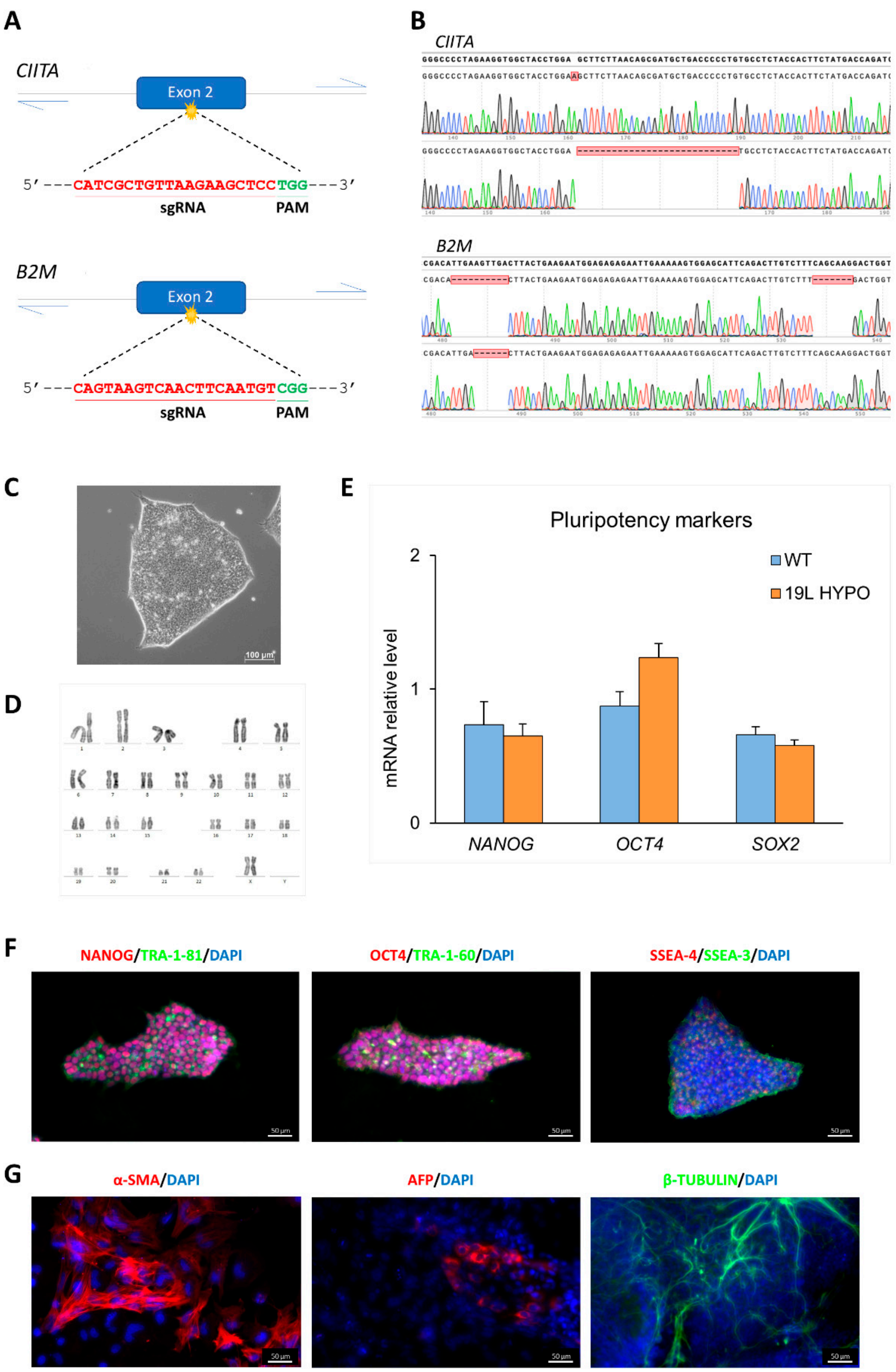

Figure S2. Characterization of the 19L HYPO iPSC line. (**A**) Schematic representation of CRISPR/Cas9 strategy to target *B2M* and *CIITA* genes. (**B**) Chromatograms showing Sanger sequencing of *B2M* and *CIITA* single alleles isolated by TOPO TA cloning of the CIITA and B2M compound heterozygous 19L HYPO clone and alignment to the WT sequence using the SnapGene software. (**C**) Bright-field image showing 19L HYPO cell morphology. Scale bar: 100 µm. (**D**) Karyotype analysis of M HYPO iPSC clone. (**E**) Analysis of transcript levels of pluripotency marker genes *OCT4*, *NANOG* and *SOX2* by qRT-PCR. The 802-30F iPSC (WT) clone was taken as reference sample. Data are expressed as the mean ± standard deviation (SD) of three independent biological experiments. (**F**) Immunostaining analysis for pluripotency markers. Nuclei were stained with DAPI. Scale bars: 50 µm. (**G**) Immunofluorescence for endodermal marker *α*-fetoprotein (AFP), ectodermal marker βIII-tubulin (β-TUBULIN) and mesodermal marker *α*-smooth muscle actin (alpha-SMA) showing iPSC trilineage differentiation potentiality. Scale bars: 50 µm.

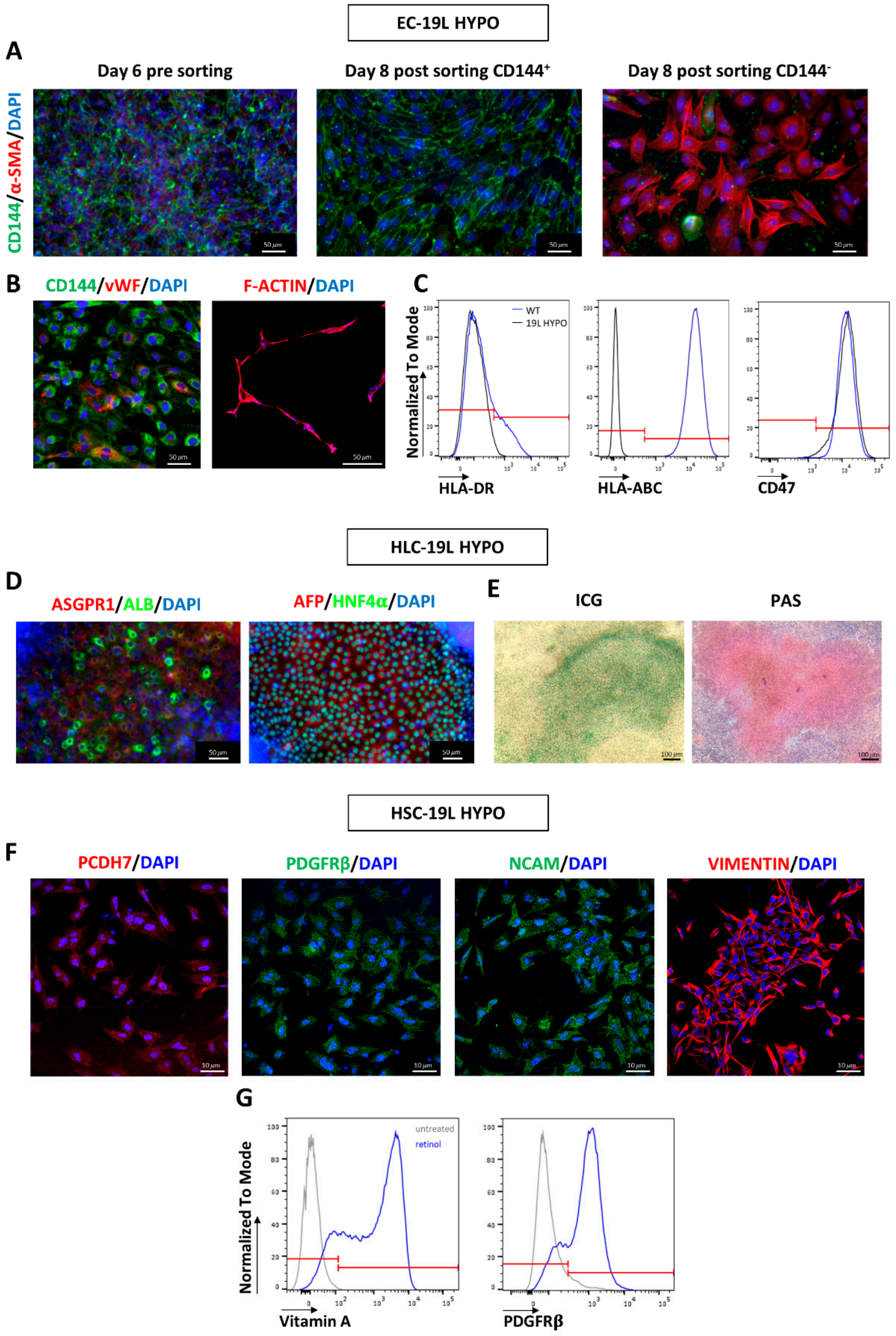

Figure S3. Analysis of 19L HYPO ability to differentiate towards endothelial-like cells (EC-19L HYPO), hepatocyte-like cells (HLC-19L HYPO) and hepatic stellate-like cells (HSC-19L HYPO). (**A**) Representative immunofluorescence images showing the expression of CD144 and α-SMA on iPSC-ECs from 19L HYPO pre- and post-sorting, (**B**) the expression of the endothelial markers CD144 and vWF (left) in EC-19L HYPO and of F-Actin in capillary-like structures formed following the in vitro tube formation assay (right). (**C**) Representative overlay histogram plots of flow cytometric analysis of HLA-DR, HLA-ABC and CD47 in IFNγ-treated endothelial cells derived from WT (black) and M HYPO cells (blue). (**D**) Representative immunofluorescence images showing the expression of the hepatocyte markers ASGPR1, ALB, HNF4α and AFP in HLC-19L HYPO cells. (**E**) Analysis of ICG uptake (left) and PAS staining showing glycogen storage (right) in differentiated cells. (**F**) Protein expression of the HSC markers PCDH7, PDGFRβ, NCAM and vimentin in HSC-19L HYPO cells

by immunofluorescence analysis. (**G**) Representative overlay histogram plots of flow cytometry analysis of iPSC-HSC PDGFRβ-positive cells and vitamin A-positive cells at the end of a 4-day retinol treatment. The red line divides negative (on the left) from positive (on the right) marker expression.

Table S1: List of primers used to amplify and sequence on-target and off-target sites

**Target**	**Sequence**
**On target: *B2M***	*B2M* gPCR F: CCCAGCCTGTCTGATACTTGT
ENSG00000166710	
chr15:44715428-44715450	*B2M* gPCR R: TGCGGCATCTTCAAACCTGA
	B2M seq F: GAGAGCCTCCAGAGAAAGGC
	B2M seq R: TGCTCAACTGCAGGGAAACT
**OT#1: *MACC1***	OT#1 gPCR F: TTCACTGTCACTTTGGGCGG
ENSG00000183742	
chr7:20170788-20170810	OT#1 gPCR R: ACTGTAGGGTCCTCACTTGC
	OT#1 seq1 F: TACTGTTGCACACCGTTCTGT
	OT#1 seq2 F: ATCCTGGCTAACACGGTGAA
**OT#2: *LINC00992/AC114945.1***	OT#2 gPCR F: TGTGCCACCTGAAAATTGCG
ENSG00000248663	
chr5:117415509-117546298	OT#2 gPCR R: GCCTCAGTAGTGCACATGGT
	OT#2 seq1 R: GGTAGAAGATCCCAACCAACTCT
	OT#2 seq2 F: TGTGCCACCTGAAAATTGCG
**OT#3: *AL034395.1-KAZN***	OT#3 gPCR F: GTGACAGTGCACGGGATAGT
ENSG00000280763	
chr1:14637585-14637607	OT#3 gPCR R: CCCGTGAACCTTCTAAGCCC
	OT#3 seq1 F: GAGAAGGGTGGGGACAAAGG
	OT#3 seq2 R: AAGGAGATCAGAGGGGAGGT
**OT#4: *LINC01493*** ENSG00000254562	OT#4 gPCR F: AAGAAAGCTCCTGTCATCCTGG
11:38646451-38686323:1	OT#4 gPCR R: GACCCCTTCTTCCCTGAAGTGTG
	OT#4 seq1 F: AAGCATACTGAGCAAAAGTGCAG
	OT#4 seq2 R: TGTTAAGAAACTGTGTGTTTGCAT
**OT#5: *RP11-21B23.1-RP11-21B23.2***	OT#5 gPCR F: CGGGGCTCCCCTCAGTAATA
ENSG00000261393	
chr16:50378641-50378663	OT#5 gPCR R: GGGAAGCACTTCTGACGCTC
	OT#5 seq1 R: CCCTTCCACGGCTCTGTAAT
	OT#5 seq2 R: GGGAAGCACTTCTGACGCTC
**OT#6: *TTC8***	OT#6 gPCR F: TGTTAATGGGACAGGACCACTC
ENSG00000165533	
chr14:88878928-88878950	OT#6 gPCR R: GCACAGAAGCCAGAAGTTACG
	OT#6 seq1 R: GCACAGAAGCCAGAAGTTACG
	OT#6 seq2 R: GCACAGAAGCCAGAAGTTACG
**OT#7: *RYR3***	OT#7 gPCR F: GAGAGACAGGGCAAAGACCC
ENSG00000198838	
15:33310962-33866121	OT#7 gPCR R: AGGCAAAACTGGAACCCACA
	OT#7 seq1 F: ACAGAAGACTTTAGCCAGTTGC
	OT#7 seq2 R: GAGGAGGTGGTGGCACTAAA
**OT#8: *SETD7***	OT#8 gPCR F: TTGTTTAACTTTGTTTACTGCAGGC
ENSG00000145391	
4:139495941-139606699	OT#8 gPCR R: ATACAGTTCCTGCAAGGATTCCA
	OT#8 seq1 F: TTGGCCCTCTTGAAATTTGGGATTA
	OT#8 seq2 R: TGGTAACGACACGCTTTGAA

Abbreviations: OT, off-target; F, forward; R, reverse; gPCR, genomic polymerase chain reaction; seq, sequencing

Table S2: List of Taqman probes and primers

Catalog number	Gene Symbol	Description
**Hs0275891_g1**	*GAPDH*	Glyceraldehyde-3-Phosphate Dehydrogenase
**Hs00742896_s1**	*Oct4*	POU Class 5 Homeobox 1
**Hs02387400_g1**	*Nanog*	Nanog Homeobox
**Hs00602736_s1**	*SOX2*	SRY-Box Transcription Factor 2
**Hs00610080_m1**	*Brachyury (T)*	T-Box Transcription Factor T

RefSeq	Gene Symbol	Sequence 5'-3'
**NM_000194.3**	Hs *HPRT*	Fwd: GGCAGTATAATCCAAAGATGGTCA<br>Rev: TCCTTTTCACCAGCAAGCTTG
**NM_022454.4**	Hs *SOX17*	Fwd: GGCGCAGCAGAATCCAGA<br>Rev: CCACGACTTGCCCAGCAT
**NM_001008540.2**	Hs *CXCR4*	Fwd: TCCATTCCTTTGCCTCTTTTGC<br>Rev: TGTCCGTCATGCTTCTCAGTT
**NM_178849.3**	Hs *HNF4α*	Fwd: ACTACATCAACGACCGCCAGT<br>Rev: ATCTGCTCGATCATCTGCCAG
**NM_001134.3**	Hs *AFP*	Fwd: AAATGCGTTTCTCGTTGCTT<br>Rev: GCCACAGGCCAATAGTTTGT
**NM_000477.7**	Hs *ALB*	Fwd: GCACAGAATCCTTGGTGAACAG<br>Rev: ATGGAAGGTGAATGTTTCAGCA
**NM_001173523.2**	Hs *PCDH7*	Fwd: TGTGGGAGCAGGAGACAACA<br>Rev: CACTCTACGAAATGGCTGTTTGC
**NM_002609.3**	Hs *PDGFRB*	Fwd: ATCAGCAGCAAGGACACCAT<br>Rev: CAGGAGAGACAGCAACAGCA
**NM_001927.4**	Hs *DES*	Fwd: CAACAAGAACAACGACGCCC<br>Rev: GGGAATCGTTAGTGCCCTTCA

Table S3: List of primary and secondary antibodies

Target Antigen	Company	Catalog Number
OCT3/4 (Octamer-binding transcription factor 3/4)	Santa Cruz	sc-5279, RRID:AB_628051
NANOG (Nanog homeobox)	Santa Cruz	sc-33759, RRID:AB_2150401
TRA-1-60 (T cell receptor alpha locus-1-60)	Millipore	MAB4360, RRID:AB_2119183
TRA-1-81 (T cell receptor alpha locus-1-81)	Millipore	MAB4381, RRID:AB_177638
SSEA-3 (Stage-specific embryonic antigen 3)	Santa Cruz	sc-21703, RRID:AB_628288
SSEA-4 (Stage-specific embryonic antigen 4)	Santa Cruz	sc-21704, RRID:AB_628289
α-SMA (α-Smooth muscle actin)	Sigma	C6198, RRID:AB_476856
AFP (Alpha-fetoprotein)	Santa Cruz	sc-51506, RRID:AB_626514
β-TUBULIN	Millipore	CBL412X, RRID:AB_1977541
CD144 (vascular endothelial-cadherin)	R&D	AF938, RRID:AB_355726
vWF (von Willebrand Factor)	DAKO	A0082, RRID:AB_2315602
ASPGR1 (Asialoglycoprotein receptor)	Santa Cruz	sc-52623, RRID:AB_667806
HNF4α (Hepatocyte nuclear factor 4 alpha)	Abcam	ab92378, RRID:AB_10562973
ALB (Albumin)	Bethyl	A80-129A, RRID:AB_67016
PCDH7 (Protocadherin 7)	Abcam	ab139274, RRID:AB_2868608
PDGFRβ (Platelet-derived growth factor receptor β)	Abcam	ab32570, RRID:AB_777165
NCAM (Neural cell adhesion molecule)	Millipore	AB5032, RRID:AB_2291692
VIMENTIN	Thermo Fisher Scientific	PA5-27231, RRID:AB_2544707
**Secondary Antibodies**		
Donkey anti-Rabbit IgG (H + L) Alexa Fluor 546	Thermo Fisher Scientific	A10040, RRID:AB_2534016
Goat anti-Mouse IgM Alexa Fluor 488	Thermo Fisher Scientific	A-21042, RRID: AB_2535711
Donkey anti-Mouse IgG (H + L) Alexa Fluor 546	Thermo Fisher Scientific	A10036, RRID: AB_2534012
Goat anti-Rat IgM, Alexa Fluor 488	Thermo Fisher Scientific	A-21212, RRID: AB_2535798
Donkey anti-Goat IgG (H + L) Alexa Fluor 488	Thermo Fisher Scientific	A11055, RRID:AB_2411589
Goat anti-Rabbit IgG (H + L) Alexa Fluor 488	Thermo Fisher Scientific	A-21206, RRID: AB_2289872
